# Changes over time in craniocerebral morphology and syringomyelia in cavalier King Charles spaniels with Chiari-like malformation

**DOI:** 10.1186/1746-6148-8-215

**Published:** 2012-11-08

**Authors:** Colin J Driver, Luisa De Risio, Sarah Hamilton, Clare Rusbridge, Ruth Dennis, Imelda M McGonnell, Holger A Volk

**Affiliations:** 1 Department of Veterinary Clinical Sciences, Royal Veterinary College, London, UK; 2 2The Animal Health Trust, Newmarket, UK; 3 Stone Lion Veterinary Hospital, Goddard Veterinary Group, London, UK; 4 Depertment of Veterinary Basic Sciences, Royal Veterinary College, London, UK

## Abstract

**Background:**

Chiari-like malformation (CM) and syringomyelia is a neurological disease complex with high prevalence in cavalier King Charles spaniels (CKCS). The natural progression of this disease with time has not been described. The objectives of this study were to i) determine if syringomyelia progresses with time ii) determine if features of craniocrebral morphology previously associated with CM are progressive (including caudal cranial fossa volume, caudal cranial fossa parenchymal volume, ventricular dimensions, height of the foramen magnum and degree of cerebellar herniation). A retrospective morphometric analysis was undertaken in 12 CKCS with CM for which repeat magnetic resonance images were available without surgical intervention.

**Results:**

The maximal syrinx width, height of the foramen magnum, length of cerebellar herniation and caudal cranial fossa volume increased over time. Ventricular and caudal fossa parenchymal volumes were not significantly different between scans.

**Conclusions:**

The results of this study suggest that syringomyelia progresses with time. Increased caudal cranial fossa volume may be associated with active resorption of the supraoccipital bone, which has previously been found in histology specimens from adult CKCS. We hypothesise that active resorption of the supraoccipital bone occurs due to pressure from the cerebellum. These findings have important implications for our understanding of the pathogenesis and variable natural clinical progression of CM and syringomyelia in CKCS.

## Background

Chiari-like malformation (CM) is a malformation of the hindbrain and the surrounding caudal cranial fossa (CCF) reported in small breed dogs. The condition is named after its analagous human counterpart, Chiari-type 1 malformation [1]. In cavalier King Charles spaniels (CKCS) the condition has a complex oligogenic trait of moderately high heritability [2-5]. CM is characterised by herniation of part of the cerebellar vermis through the foramen magnum [6,7]. Other reported abnormalities include occipital bone hypoplasia/dysplasia or a ‘shallow’ occipital bone [8], kinking of the medulla and malformations of the craniocervical junction [8-10], ventriculomegaly or hydrocephalus [11] and syringomyelia (SM) [6]. SM is a single or series of non-cerebrospinal fluid filled cavities within the spinal cord parenchyma, the formation of which is most likely associated with alteration of cerebrospinal fluid (CSF) flow [12]. SM is responsible for clinical signs of neurological disease in dogs including pain, cervical scoliosis and ataxia [6,13]. There is a high prevalence of SM in symptomatic CKCS with CM [3]. The pathogenesis of CM/SM is therefore often investigated concurrently.

The relationship between CM and SM in CKCS is thought to be compression of subarachnoid CSF pathways and alteration of CSF flow at the level of the foramen magnum [14]. In humans with Chiari-type 1 malformation, this alteration is associated with hypoplasia of the bones of the posterior fossa (analagous to the canine CCF) , with overcrowding of a normally developed hindbrain and consequential cerebellar tonsillar herniation [15]. Studies of CKCS cerebral and cranial morphology have revealed conflicting evidence regarding the association between SM and hypoplasia of the bones of the CCF, CCF volume and cerebellar herniation [7-9,11,16]. A possible shared limitation of these studies is a failure to identify an appropriate control group given that SM may be progressive; the prevalence of SM in asymptomatic CKCS scanned for breeding puposes is 25% at 12 months and 70% in dogs aged 72 months or more [17]. A subsequent study found an association between CCF volume and SM when comparing age-matched groups [18]. Changes in CCF volume with time may be an aetiological factor in CM/SM progression. A previous histological study of CCF bones from adult CKCS has found evidence of active remodelling of the supraoccipital bone, with replacement of bone with cartilage [19]. There may be a connection between this active remodelling and CCF volume if it occurs such that the supraoccipital bone thickness is reduced from within, thus increasing the inner CCF volume without changing the outer CCF dimensions.

There is a need for better understanding of the progression of CM and SM in dogs. We hypothesised that syrinx width increases with time in CKCS. In addition, we hypothesised that CCF volume, foramen magnum height and cerebellar herniation would progressively increase with time in CKCS with CM. We conducted a retrospective morphometric study of magnetic resonance images (MRI) from CKCS with CM. Analysis of craniocerebral morphometry from CKCS with CM for which two separate MRI studies were compared. Our findings suggested that when present, the radiological appearance of SM is progressive (increased syrinx width). In addition, foramen magnum height, length of cerebellar herniation and CCF volume were significantly increased. There was no significant difference for CCF parenchymal volume or ventricular system volume between the first and second scans. This suggests there may be dynamic changes to the bones of the CCF which has implications for the possible pathogenesis of CM and SM in CKCS.

## Results

Twelve CKCS were included in the study, of which eight were male (67%) and four were female (33%). The mean age when first scanned was 44.3 months ± 30.27 months. The median scan interval was 9.5 months (3 – 83 months). Ten of twelve dogs presented for the first scan with at least one clinical sign that could be attributable to CM/SM (83.3%). Presenting clinical complaints and treatments are summarised in Table 1. The majority of dogs (58%, 95% CI 31.95 to 80.67%) presenting for a second scan did so due to clinical complaints that were considered unrelated to the initial presenting condition and thus were suspected to represent a new condition. The difference in presenting complaints (attributable to CM/SM or not) between scans was significant (p=0.04). Four dogs were re-scanned due to poor control of the original complaint and one the development of dysaesthetic behavior.

**Table 1:** Summary of clinical complaint at presentation and treatments

**Case Details**		**First Scan**		**Scan interval (months)**	**Second Scan**		**Change in SM lesion width (mm)**
**No.**	**Age (months)/sex**	**Clinical complaint**	**Treatment**		**Clinical complaint**^**2**^	**Treatment**	
**1**	78/female neutered	Cervical hyperaesthesia Ataxia	Carprofen	42	Poor control of original complaint	Prednisolone	0
**2**	59/male neutered	Dysaesthetic behaviour^1^	Gabapentin	5	Poor control of original complaint	Gabepentin	1.4
**3**	13/male neutered	Dysaesthetic behaviour^1^	Gabapentin	17	Poor control of original complaint	Prednisolone	0
**4**	74/male neutered	Cervical hyperaesthesia	Gabapentin	3	Dysaesthetic behaviour^1^	Pregabalin	0.1
**5**	6/female	Dysaesthetic behaviour^1^ Ataxia	Meloxicam Gabapentin	83	Recurrent generalised seizures	Phenobarbitone Gabapentin Meloxicam	3.5
**6**	51/male neutered	Cervical hyperaesthesia	Acetazolamide Meloxicam	6	Poor control of original complaint	Acetazolamide Gabapentin	0.3
**7**	15/male	Seizures	Phenobarbitone	20	Recurrent generalised seizures	Phenobarbitone Levetiracetam	2.2
**8**	67/male neutered	Cervical hyperaesthesia Head tilt Ataxia	Clindamycin	4	Complex partial seizures	Phenobarbitone	0
**9**	24/female	Cervical hyperaesthesia	None	11	Recurrent generalised seizures	Potassium Bromide	2.7
**10**	6/male	Cervical hyperaesthesia	Carprofen	37	Migrating foreign body	Carprofen Prednisolone Trimethoprin-sulphonamide	1.4
**11**	90/male neutered	Facial paresis	Potentiated amoxcillin Meloxicam	3	Head tilt	Enrofloxacin	0.4
**12**	48/female neutered	Cervical hyperaesthesia	Gabapentin	8	Complex partial seizures	Potassium Bromide	0

Dysaesthetic behaviour included phantom scratching and facial rubbing.

Clinical complaints considered not attributable to CM/SM included seizures, head tilt, facial paresis and migrating foreign bodies.

### SM progression

**Figure 1:**
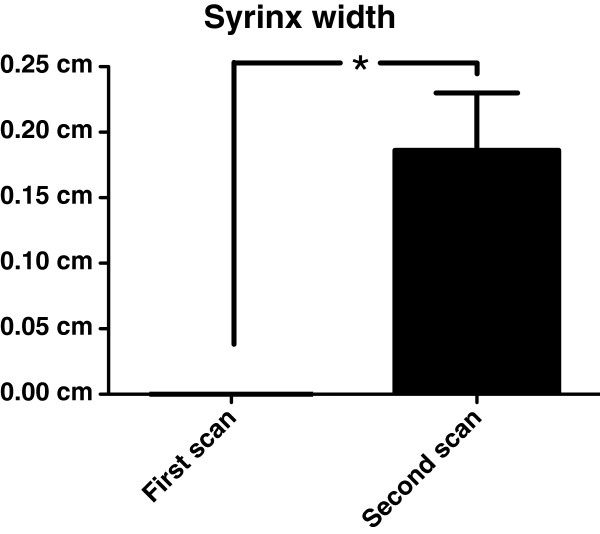
**Progression of syrinx width.** Bar graph displaying distribution of syrinx width for first and second scans. There was a statistically significant difference in syrinx maximal transverse width between first and second scans (0.08cm ± 0.14cm vs 0.18cm ± 0.16cm, p=0.01). Bar represents mean (SD), * represents *P<0.05*.

Two CKCS (17%) had syringes at the time of the first scan, this increased to five (42%) with syringes at the time of the second scan. Three dogs initially had central canal dilation (25%), which increased to four dogs (33%) following the interval. Three of five dogs re-presenting due to clinical signs attributable to CM/SM had increased SM lesion width in the scan interval (Table 1). There was a statistically significant (p=0.01) increase in SM lesion width between the first (0.08cm ± 0.14cm) and second (0.18cm ± 0.16cm) scans (Figure 1).

### Foramen magnum height and cerebellar herniation progression

**Figure 2:**
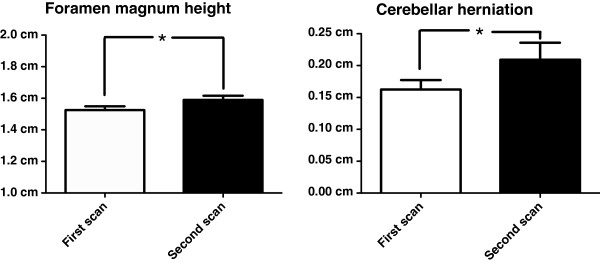
**Progression of foramen magnum height and length of cerebellar herniation.** Bar graphs displaying distribution of foramen magnum height and cerebellar herniation for first and second scans. There was a statistically significant difference between first and second scans for foramen magnum height (1.52cm ± 0.08cm vs 1.59cm ± 0.09cm, p=0.025) and length of cerebellar herniation (0.17cm ± 0.05cm vs 0.22cm ± 0.09cm, p=0.021). Bar represents mean (SD), * represents *P<0.05*.

Foramen magnum height increased in eleven (92%) dogs in the scan interval. Foramen magnum height increased significantly (p=0.025) between the first (1.52cm ± 0.08cm) and second (1.59cm ± 0.09cm) scans (Figure 2). Cerebellar herniation length increased in eight (73%) dogs in the scan interval. There was a statistically significant (p=0.021) increase in the length of cerebellar herniation between the first (0.17cm ± 0.05cm) and second (0.22cm ± 0.09cm) scans (Figure 2).

### Craniocerebral volumetric progression

**Figure 3:**
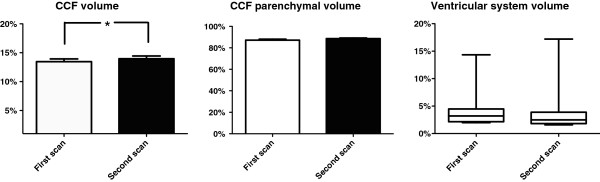
**Progression of craniocerebral volumes.** Bar graphs displaying distribution of caudal cranial fossa (CCF) volume, CCF parenchymal volume, and box and whisker plot displaying distribution of ventricular system volume between first and second scans. There was a statistically significany difference between first and second scans for CCF volume (13.46%, ± 1.45% vs 13.73% ± 1.59%, p=0.018). There was no statistically significant difference between first and second scan for CCF parenchymal volume (87.20% ± 3.09 vs 88.59% ± 1.97%, p=0.065) and the ventricular system volume (3.21%, 2.00 - 14.34 vs 2.51%, 1.602 - 17.20%, p=0.188). Bar represents mean (SD) or median (range), * represents *P<0.05*.

There was a significant difference (p=0.018) in CCF volume between the first (13.46%, ± 1.45%) and second (13.73% ± 1.59%) scans (Figure 3). There was no significant difference (p=0.188) in CCF parenchymal volume (87.20% ± 3.09 vs 88.59% ± 1.97%) or volume of the ventricluar system (p=0.188) between the first and second scans (3.21%, 2.00 - 14.34 vs 2.51%, 1.602 - 17.20%, Figure 3).

## Discussion

These results suggest that SM lesion width increases in CKCS with CM with time. Further, the height of the foramen magnum, extent of cerebellar herniation and CCF volume significantly increased with time. These findings have important implications for our understanging of the pathogenesis and natural progression of medically managed CM/SM in CKCS.

SM lesion width increased significantly in the scan interval. The number of CKCS with syringes (lesions with transverse width of greater than 2mm) increased from 2/12 to 5/12 in the scan interval. Although not all CKCS developed lesions of this width, there was progressive central canal dilation, which is a precursor of syrinx formation [20,21]. Thus this is the first report of SM progressing radiologically in CKCS with CM with time. As maximal syrinx width is associated with pain [13] this increase may also be clinically significant. Clinical progression was not determined in this study due to its retrospective nature and case selection criteria. Progression of clinical signs in medically treated CM/SM has recently been reported elsewhere [22]. Severe progression of clinical signs in our study population may have biased our case selection; CKCS with a more severe clinical phenotype prompting repeat MRI may have been more likely to have increased SM lesion width. In this study, significantly more CKCS presented for the second scan with clinical complaints considered not attributable to CM/SM.

Several studies have suggested a link between craniocerebral volumes, cerebellar herniation and the development of SM in CKCS. Reduced CCF volume as a result of impaired occipital bone development has been implicated as the cause of cerebellar herniation [6,8,9]. The adult occipital bone develops from the basioccipital, exoccipital, and supraoccipital bones which are derived from distinct somatic mesodermal derived cartilages [23]. A mesodermal insufficiency has therefore been proposed as a mechanism for mis-match between cerebral and cranial volumes and their association with SM [24], however, morphometric studies have not found this association in dogs [8,11,16]. CKCS have similar CCF volume to other small breeds such as the pug [24]. Conversely, CKCS have similar volumes of parenchyma within the CCF to larger Labrador retrievers [24]. In CKCS, small but significant increases in parenchymal volume within the CCF are associated with more severe SM [11]. Furthermore, in contrast to other small breed dogs, CKCS exhibit correlation between increased cerebellar volume and cerebellar crowding within the caudal aspect of the CCF (indicating that relative increases in cerebellar volume are more severe in the caudal aspect of the bone cavity) [25]. The findings of this study suggest that CCF volume increases significantly with time and therefore may play a role in progression of the disease.

The height of the foramen magnum was significantly increased in the scan interval. Our study also suggests the length of cerebellar herniation is increased. A positive association has previously been found between foramen magnum size and cerebellar herniation in CKCS [9]. This may represent a dynamic change occuring to the occipital bones that form the foramen magnum in response to the previously discussed parenchymal overcrowding of the CCF. This is supported by the previous finding that a large part of the supraoccipital bone of adult CKCS is cartilage, suggesting active remodelling [19]. We hypothesise that the increased CCF volume may be a result of inner resorption of the overlying occipital bones rather than an increase in its dimensions. The occipital bones in adults and children show a resorbtive pattern of the bone around the cerebellar hemispheres [26]. Bone remodelling occurs as an adaptation to mechanical load according to Wolff’s law. The pulsatile movements of the cerebellum occuring during systole could exert a mechanical pressure on the caudal aspect of the CCF leading to occipital bone resorption, hence increasing the height of the foramen magnum and possibly changes in the extent of cerebellar herniation. In support of this theory, intra-operative and post-mortem findings of CKCS with CM have revealed that the supraoccipital bone overlying the cerebellar vermis can be remarkably thin and sometimes eroded so that the foramen magnum is enlarged dorsally [27]. A thick cartilagenous band is frequently found in surgery in place of the supraoccpital bone [C. Rusbridge, personal communication]. Importantly, the reduced cerebrospinal flow velocity as a result of CCF remodelling may alter the rate of progression of SM, which may explain the high prevalence of asymptomatic SM in CKCS and the variable response to medical treatment.

An alternate mechanism for the failure of adaptation of the CCF in CKCS previously proposed is early closure of bone sutures, similar to craniosynostosis in children [24]. Early suture closure may affect the jugular foramina enclosing the inferior petrosal and sigmoid sinus, resulting in increased intracranial pressure and/or reduced resorption of CSF such as occurs in children [28]. This is a proposed mechanism for the development of SM in the Griffon Bruxellois that do not display cerebellar herniation [29].

There are limitations to the measurements made in this study. Firstly, it should be noted that variations in cerebellar herniation may occur throughout the cardiac cycle which were not accounted for. However, as noted in previous human studies, this limitation should be relatively minor and the variation is unlikely to significantly affect our measurements or conclusions [30]. Secondly, cerebellar herniation is increased by flexion of the neck [31]. The effect of positioning was minimised in this study. Some medications used to treat CM/SM, including corticosteroids, may influence the progression of CM/SM on MRI by altering cerebrospinal fluid production. None of the dogs in this study received corticosteroids in the scan interval (Table 1). The medications prescribed were not considered likely to affect our measurements.

## Conclusions

CCF volume, foramen magnum height, length of cerebellar herniation and SM lesion width increased significantly over time. The CCF parenchymal volume and ventricular volume did not change significantly. This enhances our understanding of the pathogenesis of CM/SM in CKCS. We hypothesis that dynamic remodelling may occur to the occipial bone. This could explain the varied phenotype of the disease.

## Methods

### Subject selection

Clinical database software of three institutions was retrospectively reviewed between May 2004 and May 2010 (Royal Veterinary College, Animal Health Trust and the Stone Lion Veterinary Hospital). CKCS were selected for inclusion if they had radiological findings consistent with CM (indentation of the cerebellum and herniation of the cerebellar vermis into the foramen magnum) that underwent two cranial MRI studies, both of which included sagittal and transverse slices from the cribiform plate rostrally to the third cervical spinal cord segment caudally. For each case selected, the presence of CM was confirmed by a European neurology or diagnostic imaging specialist. CKCS with a concurrent diagnosis of space occupying lesions or lesions associated with raised intracranial pressure were not included.

CKCS that were included in the study were assigned a case number. Descriptive data recorded included patient sex, age and clinical complaint at first and second presentation for MRI scan. The scan interval and treatments were additionally recorded and described. MRI scans were randomly assigned an image number such that analysis could be blinded to all descriptive data including whether it was the first or second scan. MRI scans were subsequently grouped into first and second scans prior to statistical analysis. A Fishers exact test comparing the presenting complaints (attributable to CM/SM or not) was performed between the two groups.

### Magnetic resonance image analysis

All dogs in this study had been positioned for MRI in dorsal recumbency with the cervical spine in an extended position. The presence of SM was assessed using sagittal and transverse T2-weighted MRI of the cervical spinal cord. Only the cervical spinal cord was assessed as SM most commonly affects this region [32]. SM was defined as well-demarcated intramedullary lesions associated with the central canal, hyperintense on T2 weighted and hypointense on T1 weighted images. Lesions greater than 2 mm diameter in transverse plane were described as syringes. Lesions of 0–2 mm diameter were described as central canal dilation. For statistical analysis, the maximal transverse width of the lesion (SM lesion width) was recorded using commercially available imaging software (OsiriX Medical Imaging Software 3.8.1) using a technique previously described [13].

Cerebellar herniation length was assessed from mid-sagittal T2 weighted images using the same imaging software, with a previously described technique [9]. Foramen magnum height was first assessed by measuring a line from the most dorsal aspect of the basioccipital bone to the ventral most aspect of the supraoccipital bone. Cerebellar herniation length was measured from the tip of the cerebellar vermis to the point of bisection of the line used to measure foramen magnum height [9].

Craniocerebral volumetric data was obtained using a method previously described in detail [11,18,24]. Transverse T2-weighted images were exported to a comercially available 3D modelling software programme (Mimics® 13.10, Materialise n.v., 2009). The cranial fossa was divided into the rostral/middle cranial fossa and the caudal cranial fossa by the tentorium cerebelli. Following manual tracing of a series of two-dimenstional masks from individual slices [11,18,24], the following were calculated by the software:

CCF volume, expressed as a percentage of total cranial fossa volume

CCF parenchymal volume, expressed as a percentage of total cerebral parenchymal volume

Ventricular system volume, expressed as a percentage of the total cerebral parenchymal volume

Individual masks were drawn by the same observer (SH).

### Statistical analysis

Commercially available statistical software was used for data analysis (Prism® for Windows Version 5.00, Graphpad Software Inc, 2007). Data sets were assessed for normality of distribution with the Shapiro-Wilk W test. Where data sets for the two groups were normally distributed, a paired one-tailed t-test was used to analyse the statistical significance of data. Where data sets were not normally distributed, a one-tailed Wilcoxon signed rank test was used. One-tailed tests were used considering our prediction of the direction of effect based on the described observations of changes to the supraoccipital bone and the general assumption that SM is a progressive disease.

Parametric data is presented as the mean ± the standard deviation and non-parametric data is presented as the median with range. A p value <0.05 was considered significant.

## Competing interests

The authors declare that they have no competing interests.

## Authors‘ contributions

CJD assisted with the morphometric analysis, performed the statistical analysis and drafted the manuscript. SH performed the morphometric analysis. LDR, RD and CR participated in the design of the study and recruited cases. IMM and HAV conceived of the study, participated in its design and coordination and helped to draft the manuscript. All authors read and approved the final manuscript.
